# Conceptual and Biofunctional Embodiment: A Long Story on the Transience of the Enduring Mind

**DOI:** 10.3389/fpsyg.2016.01990

**Published:** 2017-01-10

**Authors:** Asghar Iran-Nejad, Auriana B. Irannejad

**Affiliations:** ^1^Department of Educational Studies in Psychology, Research Methodology, and Counseling, The University of Alabama, TuscaloosaAL, USA; ^2^Department of Psychology, The University of Alabama at Birmingham, BirminghamAL, USA

**Keywords:** biofunctional understanding, conceptual understanding, embodied understanding, ongoing biofunctional activity, momentary constellation firing, ease of understanding dimension, collective organization, collective announcement

## Abstract

We examine how embodiment in biological activity is different from conceptual embodiment as reflected in classic, modern, and postmodern perspectives on tacit knowledge. The central theme of the essay is how understanding is embodied conceptually and biofunctionally. We focus (a) on how biofunctional understanding (BU) is different from conceptual understanding (CU) and (b) on how the overall differences between these two types of embodied understanding are complementary. We show here from a conceptual perspective that embodiment theories have diverged on the meaning of embodiment; but convergence may be more likely across future perspectives if we first redefine the construct of tacit knowledge as tacit understanding and then define (explicit) CU as being directly grounded in tacit understanding, for the purpose of comparison with BU defined as being grounded in biological activity. We illustrate the complementary differences between conceptual and biofunctional embodiment of understanding first in the absence of language and then using a particular statement format and the implicit analogy of biofunctional embodiment in other bodily systems. We conclude with a suggestion about the directly uncovered but highly related embodiment of language in a section on future research.

## Transition to Second Generation Cognitive Science

The inability of the abstract schemas to account for understanding may have been one of the reasons why researchers grew dissatisfied with the formalism of the first generation cognitive science. In the late 1970s, the field experienced a critical transition from the first to second generation cognition. According to Neisser (1976), information processing had momentum and prestige but lacked in commitment to human nature. Accordingly, almost as if overnight, the rapidly growing field of cognition turned controversial over structural permanence (Iran-Nejad, 1980/1987) and other static features of long-term memory schemas (Black and Wilensky, 1979; Thorndyke and Yekovich, 1980; Posner et al., 1982; Wilensky, 1983; Brewer and Nakamura, 1984). Mental schemas were debated from every conceivable angle including meaning leanness (Anderson et al., 1978; Alba and Hasher, 1983), neglect of affect (Zajonc, 1980), symbol grounding (Searle, 1980; Harnad, 1990), inflexibility (Iran-Nejad et al., 1981/1984), connectionist-network infrastructure (Minsky, 1980), decontextualization (Brown et al., 1989), and, finally, disembodiment (Clark, 1998).

A retrospective look at the above transitional literature suggests two remarkable considerations. First, the literature, especially the symbol-grounding literature, reads like being deeply concerned about understanding without using the term *understanding*. Second, some leading investigators were groping like they had experienced a sudden enlightenment or even disillusion that their already popular first generation cognitive science was no longer worthy of their attention and that they were better off with some other equally popular replacement. Thus, Anderson spoke of a radical shift in his schema-instantiation theory from favoring extreme abstractness (Anderson, 1977) to a later emphasis on richness of exemplars (Anderson, 1984). Similarly, Rumelhart introduced a similarly radical shift from his symbolic story grammar or his theory of monolithic schemas (Rumelhart, 1975, 1980; Iran-Nejad et al., 1992; Do and Rahm, 2007) to a new focus (see, especially, Rumelhart, 1984, conference paper) on subsymbolic parallel distributed processing (PDP) connectionism (Rumelhart and McClelland, 1986). By the same token, to cite one other example, elaborating on their conceptual change theory (Posner et al., 1982), Strike and Posner (1992) stated that they “were interested in a phenomenon that is analogous to Kuhn’s notion of a paradigm shift” (p. 148). The fact that these pioneering scholars turned away from their already influential work is evidence for a sudden enlightenment; and the fact that they each headed in a different direction in search of something revolutionary like PDP connectionism may be taken as evidence for the lack of direction about where the field was better off heading.

## Knowledge Schemas and Understanding

Two lines of inquiry focused directly on understanding during the transition period. One of these was by Black and Wilensky (1979) who suggested that the abstract story schema may be a consequence of story understanding rather than a prerequisite cause for it. Focusing on Rumelhart’s (1975) story-grammar, these researchers reasoned that the structure of a story may presuppose understanding the story such that “there is no reason to determine the structure because we must have understood the story before we can discover the structure” (p. 228). The second line of research on surprise-ending story understanding was derived from biofunctional theory (Johnson, 2015). Using the vivid analogy of the camera as the prerequisite system and the pictures it takes as its postfunctional product, Iran-Nejad (1980/1987) made the same point about the irrelevance of abstract structures as made by Black and Wilensky (1979), explaining that the structure of the pictures a camera takes is unlikely to tell us anything about *how* the camera takes pictures—without using picture templates. Later, Rumelhart (1984) explained how schemas may emerge from subsymbolic “neural networks” but did not discuss how subsymbolic neuron-less networks understood stories (Wilensky, 1983; Iran-Nejad, 1989).

## The Rise of Embodied Cognition

The transition period exposed a number of problems with abstract schema theories, chief among which being the need for a solution to the symbol-grounding problem. In the 1990s, cognition researchers used the occasion of this transition to introduce embodiment for a solution. The demand for the new solution was widespread; and before long embodied cognition swept the planet (Wilson, 2002; Adams, 2010). Making the embodiment solution to fit new problems demanded a working definition for the term *embodiment*. Without a satisfying definition, embodiment could not be a solution to symbol grounding or any of the other problems raised during the transition period. Again, scholars followed divergent paths in search of a definition none of which touched upon embodied understanding (Johnson, 2015), as demonstrated in three reviews of the literature by Wilson (2002), Kiverstein (2012), and Gärtner (2013). Wilson (2002) identified six definitions for the term. Of these, four she evaluated as the least-partially true, one as most problematic, and one as the least explored but most promising. A decade later, Kiverstein (2012) wondered exactly what researchers meant when they said that cognition is embodied, described the enterprise in terms of four E’s (embodied, embedded, extended, enacted) and one A (affective), matching, like Wilson (2002), rather closely the diversity of the transition era. At about the same time, Gärtner (2013) distinguished another six different meanings, some he saw as promising but each standing alone waiting for elusive integration. In the meantime, the gaps between internal knowledge representations and conceptual understanding (CU), on the one hand, and between knowledge-enabled understanding and biology-enabled understanding, on the other, proved much tougher than the cognitive revolution of the 1960s had initially anticipated (Bransford and Johnson, 1972; Bobrow and Collins, 1975).

## Tacit Knowledge and Tacit Understanding

Readers may be able to discern some semblance of unity in diversity surrounding a definition for embodiment grounded in tacit knowledge—knowledge that cannot be directly told or verbalized. Related to the present discussion, a particularly readable essay on tacit knowledge is a commentary by Prawat (2000) on Iran-Nejad’s (2000) biofunctional-understanding theory. According to this theory, (a) the hallmark of a genuine event of understanding is the *extraordinary click of understanding* (ECOU, let us say echo) we experience inside at varying levels of strikingness and (b) the hallmark of the ECOU itself is a paradox of a missing “how” function. According to Iran-Nejad (2000):

Consider, for example, the constructive learning metaphors *I know* or *I understand.* They each signify the end result of some unpacked internal process. It is meaningful to say “I know” or “l understand,” especially if the object is understood. However, it is not so meaningful to say, “I know how to know” or “I know how to understand” (Iran-Nejad, 1978). The reason we cannot make the latter statements is that we know nothing about the underlying process by which we come to know or understand something. (p. 69).

We “know-that” we know and understand without, paradoxically, “knowing-how” we know and understand. To learn more about this paradox (Iran-Nejad and Bordbar, 2013), let us assume that there is a collective “how” organizer function or, rather, a set of “what, how, why, when, where, who, and which” organizer functions “underlying” (or perhaps “under-standing”) the ECOU as a multiple-source capability. Iran-Nejad (2000) proposed that the physical living *system of subsystems and microsystems* (i.e., *neurons*) inside the skin of the understander could directly be the collective multiple-source organizer of the ECOU, implying essentially that the paradoxical ECOU is directly biofunctional in nature. More to the point are the facts (a) that the ECOU makes its entrance into cognition totally unannounced, (b) that the set of collective-organizer functions of the ECOU is missing in cognition in the sense that cognition has no idea whatsoever about the black box that produces the surprising ECOU when the latter makes its eventual appearance, and (c) that the ECOU delivers with it some exciting surprises of its own in terms of ideas and affects. These facts point to the conclusion that the set of collective multiple-source organizer functions must comprise a dynamic living system made for the extraordinary capability to be most aptly called biofunctional understanding (BU; Johnson, 2015). In his commentary, Prawat (2000) did not dispute this conclusion about the nature and relevance of the paradoxical ECOU solution (Iran-Nejad, 2013). Rather, he stated:

lran-Nejad illustrates this problem with a simple comparison. Thus, he writes, “The statements ‘*I know how to elaborate*’ and ‘*l know how to understand*’ have different effects on people’s intuitive judgments of meaningfulness” (p. 70). The fact that we do not know how we understand but we do know when we understand—the “extraordinary click” referred to by lran-Nejad—points to a process that lies beneath that of symbolic manipulation (symbols chasing symbols). The lack of awareness of how understanding occurs, coupled with the phenomenological certainty that it does occur, is prima facie evidence that the process takes place somewhere in addition to, if not other than, the symbolic or propositional level. (p. 90).

In his perceptive commentary, Prawat (2000) endorsed the biofunctional-understanding solution and recommended broadening the problem to engage tacit-knowledge proponents among first-generation modernists like Reber and colleagues (Allen and Reber, 1980; Reber, 1989), transition era subsymbolists like Rumelhart (1984), second generation postmodernists like Lakoff and Johnson (1999), as well as their classic inspirers like Polanyi (1968), Peirce (1898), Dewey (1925/1981), and Wittgenstein (1963). In the process, it may turn out that the directly inarticulable BU supports a more precise construct of tacit understanding than the more popular concept of tacit knowledge.

## The Biofunctional Understanding Spiral

Two independent lines of research (see, however, Maturana, 1978 cited in Iran-Nejad and Ortony, 1984) have directly addressed biological embodiment. One, introduced by Maturana and colleagues, focused on the concept of autopoiesis (Maturana and Varela, 1980) and evolved into the literature on the embodied mind (Maturana and Varela, 1987; Varela et al., 1991, 1999). The other was introduced at about the same time by Iran-Nejad and his colleagues and has developed into BU (Alverson, 2015) and related areas like interest (Iran-Nejad, 1987), affect (Diener and Iran-Nejad, 1986), and self-regulation (Iran-Nejad and Chissom, 1992). In this section, we describe how conceptual or knowledge-enabled understanding and biofunctional or biology-enabled understanding are different and complementary in their roles in the overall spiral of understanding.

Iran-Nejad (2000) illustrated how the ongoing understanding by a grazing prey animal that the environment is safe even amidst surrounding predators interacts with momentary knowledge in the absence of symbolic knowledge. The predator in wait also has her own understanding of how safe the meal is as long as there is no knowledge tipoff. So far there is no need to assume prerequisite knowledge for understanding on the part of either one of animals and there is all the reason to assume that understanding is the prerequisite for knowledge on the part of both. Thus, “the well-camouflaged predator may be completely safe right in front of the eyes of the prey. But as soon as its spots shift, it has already lost its meal” (p. 81) to the momentarily conceived knowledge and the new understanding by the prey that the area is unsafe.

**FIGURE 1:**
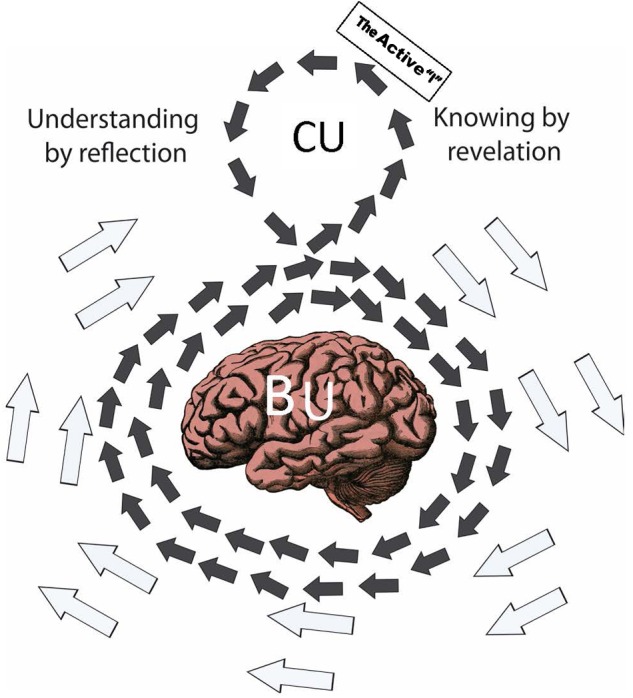
**Iran-Nejad’s spiral of biofunctional understanding (BU).** Dark arrows circulating in the shape of the number 8 represent the biofunctional spiral of understanding. Clockwise arrows represent the collective organization of ongoing biofunctional activity (OBA) and counterclockwise arrows represent momentary constellation firing (MCF) in neurons. The full OBA–MCF spiral portrays two kinds of understanding. BU happens, without any “know-how” whatsoever on the part of the understander, in the nervous and bodily systems only. Clockwise arrows separating their path to rise upward and to the right, again without any “know-how” whatsoever by the understander, represent a type of OBA-fueled MCF in neurons that “*collectively announce*” knowing by revelation in which both cognition and affect emerge together. Dark arrows turning counterclockwise—and moving forward under the knowing-how influence of the active “I” (or executive function, EF)—represent understanding by active refection on the part of the EF. “Knowing” as defined here is none other than MCF-conceived self-awareness. Counterclockwise arrows sinking to turn clockwise at the top left represent reflection-caused conceptual understanding (CU). The sharp image of the brain is meant to represent an integrated system of nervous and bodily subsystems and microsystems (i.e., neurons) not an isolated brain, nervous system, or body. Copyright 1980… 2016 by Asghar Iran-Nejad. Wholetheme Education Project. Image from Iran-Nejad, A. (2016). “Writing as a body-mind performance learning activity for educational development of wholetheme professional Artistry,” in *Writing for Professional development*, eds G. Ortoleva, M. Betrancourt, P. Tynjala, and S. Billett (Leiden: Brill), 61–87.

In **Figure 1**, dark arrows moving clockwise represent ongoing biofunctional activity (OBA) of understanding as it happens in the alertly grazing animal over time. Multiple sources contribute perpetually to OBA and knowing is but one of those sources. Another source is the active “I” of the animal. Among other contributing sources to BU are diverse sensory modalities and other internal subsystems of the body (e.g., for hunger, thirst, fear, joy) all contributing perpetually to the OBA. The OBA collectively organizes contributions from multiple sources as the collective how, why, when, which, who, and what of these sources remain biofunctional secrets.

The dark clockwise arrows rising suddenly upward to the right represent momentary constellation firing (MCF) in already alert neurons. MCF collectively announces revelatory knowledge and excitement (knowing by revelation in the figure). This happens when the shift in predator spots, as one source, alarms the already alert animal in the form of a momentary conception or revelation. Again, the how, when, … of such collective announcements are biofunctional secrets. What is important to know is that they happen, do so as portrayed in the circulating arrows, and do so in exquisite regularity.

The counterclockwise arrows moving leftward in **Figure 1** represent knowing by reflection. This is when the already alarmed animal (i.e., the active first-person of the “I”) considers by keeping the arrows moving deliberately leftward to ascertain the safety of the area. Finally, counterclockwise arrows sinking on the left to turn clockwise represent CU by reflection. This is when the prey animal reaches an understanding if the area is safe or unsafe and the predator reaches the understanding if the meal is safe or lost. And the social spiral continues perpetually in both animals in different stories.

To illustrate how knowledge is a cause and consequence of BU, let us start by examining the first-person statement *I know that I keep my body warm*. This ambiguous statement may be disambiguated in two ways in order to show grounding of the symbols either in CU (see **Figure 1**) as in CU1 *I know that I keep my body seasonally warm* or in BU as in BU1 *I know that I keep my body* about 98.6° *warm*. As their labels imply, there are some noteworthy differences in understanding between CU1 and BU1. With CU1, the active “I” can literally claim, as well as stand by this claim, the skill of knowing-how to keep the body warm as illustrated in the true CU2 *I know how to keep my body seasonally warm*. By contrast, with BU1, the active “I” can make the claim, more or less metaphorically, without being able to stand by it as illustrated by the false BU2 *I know how to keep my body about 98.6° warm.* BU2 is only metaphoric because, despite the claim, neither the first-person of the active “I” nor the third-person of anyone else, other than the body itself, knows how to keep the body 98.6° warm—as of yet, only biology and the almighty, so to speak, know how to do that (compare, also, CU3 with BU3 and CU4 with BU4—not shown). CU3 *I know that I keep my body seasonally warm and I know how to keep my body seasonally warm*, e.g., by staying on the path of migration (**true, internally coherent, embodied in CU know-how**). BU3 *I know that I keep my body about 98.6° warm and I know how to keep my body about 98.6° warm* (**false, internally incoherent, not embodied in CU know-how, embodied in BU**). Thus, for the grazing prey, the understandings—that (a) the grazing ground is safe and (b) the temperature is right—are collectively organized in the same way; but as soon as the predator spots shift or the temperature changes, the active “I” of the prey “knows-how” to decide based on moment-by-moment “knowing-that” if and when it is time to move on or to stay put. CU4 *I know that I keep my body seasonally warm* even though *I do not really know myself how to keep my body seasonably warm* **(false, internally incoherent, embodied in CU know-how)**.

## A New Perspective on the Transience of the Enduring Mind

This “new perspective” was the theme encapsulated in the title—“The schema: A structural or a functional pattern”—of a technical report first published and widely circulated in 1980 by the Center for the Study of Reading, University of Illinois at Urbana-Champaign. A lot has happened in the interdisciplinary field of cognitive science in the almost four decades since. Nevertheless, there is little risk in calling the perspective new and use the still oven-fresh theme of transience to address how the similarities and differences between CU and BU make them complementary. The answer lies in the simultaneously transient and enduring way the two interact in the spiral of BU. Represented by clockwise-moving arrows, BU is the prerequisite ongoing source for sounding CU clicks (or ECOUs) in the form of knowing by revelation (see rising clockwise arrows in **Figure 1**). Similarly, CU is a momentary cause of knowing by reflection, clockwise arrows turning counterclockwise. Knowing is a momentary prerequisite for BU by reflection, counterclockwise arrows turning clockwise. In this way, BU is an enduring contributor to OBA which is an enduring contributor to MCF, and the spiral continues but for one exception: momentary knowing by reflection must be regulated forward by the active “I” with prerequisite know-how to cause BU, which accounts for the difference between the above CU and BU statements. In this fashion, the dynamic or spontaneous and active or deliberate spiral of understanding continues its perpetual course.

## A Future Research Direction

A relevant topic here is the role of language in the BU spiral. An intriguing concept we are finding useful is a hypothesized biofunctional ease of understanding dimension to account for the order inherent in developmental progression as it happens in Piaget’s four stages of child development. This ease of understanding dimension, defined as an OBA-MCF relationship, can be more readily described with a metaphor in the limited space of this essay. We can think of MCF as a biofunctional propeller that pulls forward the OBA in proportion to the unity-in-diversity fuel it can draw from it to collectively announce rich postfunctional revelations in varying degrees of strikingness (Iran-Nejad and Stewart, 2010). In their psycholinguistic studies of language embodiment, Anna M Borghi, Claudia Scorolli, and colleagues (Scorolli et al., 2011; Borghi et al., 2013), among others, are developing methodology that lends itself remarkably well to ease of understanding (or processing) studies.

## Author Contributions

AI drafted the article first. ABI reviewed and revised. This cycle repeated for several drafts until the manuscript reached the submitted draft.

## Conflict of Interest Statement

The authors declare that the research was conducted in the absence of any commercial or financial relationships that could be construed as a potential conflict of interest.

The reviewer HS and handling Editor declared their shared affiliation, and the handling Editor states that the process nevertheless met the standards of a fair and objective review.
